# Diseases of Metabolism and Nutrition

**Published:** 1903-06

**Authors:** 


**Diseases of Metabolism and Nutrition.** By Prof. CARL VON NOORDEN. Authorised American Edition. Translated under the direction of BOARDMAN REED, M.D. Part I.—*Obesity: the Indications for Reduction Cures.* Pp. 59. Part II.—*Nephritis.* Pp. 112. New York: E. B. Treat & Company. 1903.

The first of these books deals succinctly and sensibly with the indications for reduction cures in obesity. The advice given is based on scientific principles, and is eminently characterised by its practical character and common sense. A series of sections deal with obesity when it occurs as a complication of other diseases: perhaps the section on obesity in connection with diseases of the circulatory system is especially valuable. The book is quite short, and deals with first principles only; but we think every medical man who has mastered its contents will be better able to give sound advice to his obese patients. To many its value would have been increased by a second part dealing with the details of reduction cures, but this subject is apparently to be discussed in a later volume.

The second volume, on *Nephritis*, we have read with much profit. Although longer than the first, it is quite a small book, but contains a quantity of information. The routine and rule-of-thumb methods of treatment of kidney diseases, still too much in vogue, are here shown to be false in principle and disastrous in practice. The author especially points out that the exclusive milk diet has been carried much too far, that milk in too large quantities in Bright's disease is injurious to the kidneys, and that discrimination in diet so as to adapt it to the conditions present in different forms and stages of kidney disease is essential to success in treatment. He strongly advocates the restriction of fluids in acute nephritis, and, under certain conditions, in contracted granular kidney; at other times he agrees with the administration of water freely, and gives clear indications, based on careful observations, for this mode of treatment. Another common mistake is here pointed out, that is, the continuation of vapour baths after œdema has disappeared and there is free diuresis. Further, he shows that the common belief that white meat is less injurious than red has no clinical facts to support it. This book can be strongly recommended; no one can fail to learn something from it, and it is evidently the outcome of a wide experience, mature study, and a sound judgment.

Pages 60 and 68 are misplaced. The translation of both volumes is only very moderate.

